# Identification of fusion genes in breast cancer by paired-end RNA-sequencing

**DOI:** 10.1186/gb-2011-12-1-r6

**Published:** 2011-01-19

**Authors:** Henrik Edgren, Astrid Murumagi, Sara Kangaspeska, Daniel Nicorici, Vesa Hongisto, Kristine Kleivi, Inga H Rye, Sandra Nyberg, Maija Wolf, Anne-Lise Borresen-Dale, Olli Kallioniemi

**Affiliations:** 1 Institute for Molecular Medicine Finland (FIMM), Tukholmankatu 8, Helsinki, 00290, Finland; 2 Medical Biotechnology, VTT Technical Research Center of Finland and Turku Center for Biotechnology, Itäinen Pitkäkatu 4C, Turku, 20520, Finland; 3 Department of Genetics, Institute for Cancer Research, Oslo University Hospital Radiumhospitalet, Ullernchausseen 70, Oslo, 0310, Norway; 4 Institute of Clinical Medicine, University of Oslo, PO Box 1171 Blindern, Oslo, 0318, Norway

## Abstract

**Background:**

Until recently, chromosomal translocations and fusion genes have been an underappreciated class of mutations in solid tumors. Next-generation sequencing technologies provide an opportunity for systematic characterization of cancer cell transcriptomes, including the discovery of expressed fusion genes resulting from underlying genomic rearrangements.

**Results:**

We applied paired-end RNA-seq to identify 24 novel and 3 previously known fusion genes in breast cancer cells. Supported by an improved bioinformatic approach, we had a 95% success rate of validating gene fusions initially detected by RNA-seq. Fusion partner genes were found to contribute promoters (5' UTR), coding sequences and 3' UTRs. Most fusion genes were associated with copy number transitions and were particularly common in high-level DNA amplifications. This suggests that fusion events may contribute to the selective advantage provided by DNA amplifications and deletions. Some of the fusion partner genes, such as *GSDMB *in the *TATDN1-GSDMB *fusion and *IKZF3 *in the *VAPB-IKZF3 *fusion, were only detected as a fusion transcript, indicating activation of a dormant gene by the fusion event. A number of fusion gene partners have either been previously observed in oncogenic gene fusions, mostly in leukemias, or otherwise reported to be oncogenic. RNA interference-mediated knock-down of the *VAPB-IKZF3 *fusion gene indicated that it may be necessary for cancer cell growth and survival.

**Conclusions:**

In summary, using RNA-sequencing and improved bioinformatic stratification, we have discovered a number of novel fusion genes in breast cancer, and identified *VAPB-IKZF3 *as a potential fusion gene with importance for the growth and survival of breast cancer cells.

## Background

Gene fusions are a well-known mechanism for oncogene activation in leukemias, lymphomas and sarcomas, with the *BCR-ABL *fusion gene in chronic myeloid leukemia as the prototype example [1,2]. The recent identification of recurrent *ETS*-family translocations in prostate cancer [3] and *EML4-ALK *in lung cancer [4] now suggests that fusion genes may play an important role also in the development of epithelial cancers. The reason why they were not previously detected was the lack of suitable techniques to identify balanced recurrent chromosomal aberrations in the often chaotic karyotypic profiles of solid tumors.

Massively parallel RNA-sequencing (RNA-seq) using next-generation sequencing instruments allows identification of gene fusions in individual cancer samples and facilitates comprehensive characterization of cellular transcriptomes [5-11]. Specifically, the new sequencing technologies enable the discovery of chimeric RNA molecules, where the same RNA molecule consists of sequences derived from two physically separated loci. Paired-end RNA-seq, where 36 to 100 bp are sequenced from both ends of 200 to 500 bp long DNA molecules, is especially suitable for identification of such chimeric mRNA transcripts. Whole-genome DNA-sequencing (DNA-seq) can also be used to identify potential fusion-gene-creating rearrangements. However, only a fraction of gene fusions predicted based on DNA-seq is expected to generate an expressed fusion mRNA, making this approach tedious to discover activated, oncogenic fusion gene events. In contrast, RNA-seq directly identifies only those fusion genes that are expressed, providing an efficient tool to identify candidate oncogenic fusions.

In breast cancer, recurrent gene fusions have only been identified in rare subtypes, such as *ETV6-NTRK3 *in secretory breast carcinoma [12] and *MYB-NFIB *in adenoid cystic carcinoma of the breast [13]. Here, we demonstrate the effectiveness of paired-end RNA-seq in the comprehensive detection of fusion genes. Combined with a novel bioinformatic strategy, which allowed >95% confirmation rate of the identified fusion events, we identify several novel fusion genes in breast cancer from as little as a single lane of sequencing on an Illumina GA2x instrument. We validate the fusion events and demonstrate their potential biological significance by RT-PCR, fluorescence *in situ *hybridization (FISH) and RNA interference (RNAi), thereby highlighting the importance of gene fusions in breast cancer.

## Results

### Criteria for identification of fusion gene candidates

**Figure 1:**
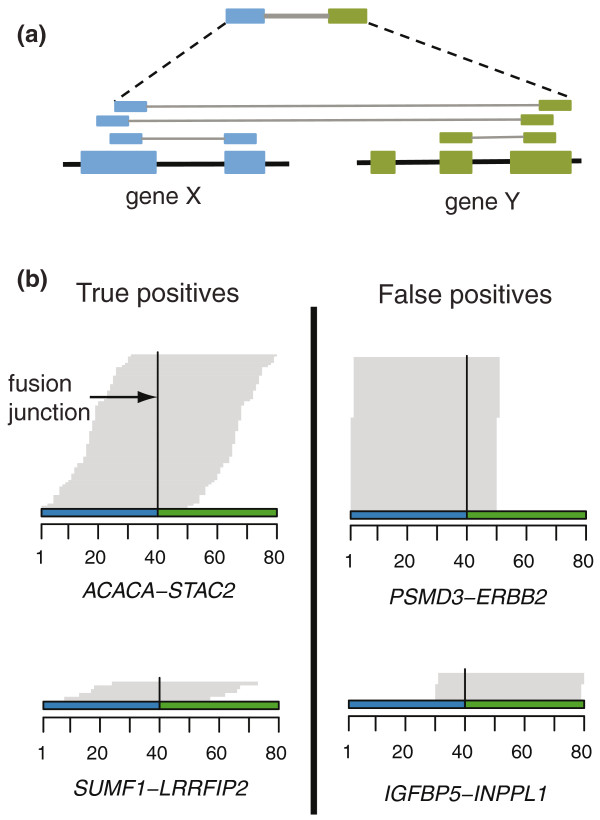
**Fusion gene identification by paired-end RNA-sequencing**. **(a) **Identification of fusion gene candidates through selection of paired-end reads, the ends of which align to two different and non-adjacent genes. **(b) **Identification of the exact fusion junction by aligning non-mapped short reads against a computer generated database of all possible exon-exon junctions between the two partner genes. Separation of true fusions (left) from false positives (right) by examining the pattern of short read alignments across exon-exon junctions. Genuine fusion junctions are characterized by a stacked/ladder-like pattern of short reads across the fusion point. False positives lack this pattern; instead, all junction matching short reads align to the exact same position or are shifted by one to two base pairs. Furthermore, this alignment is mostly to one of the exons.

To detect fusion genes in breast cancer, we performed paired-end RNA-seq using cDNA prepared from four well-characterized cell line models, as well as normal breast, which was used as a control. Between 2 and 14 million filtered short read pairs were obtained per sample for each lane of an Illumina Genome Analyzer II flow cell (Additional file 1). We discarded all fusion candidates consisting of two overlapping or adjacent genes as likely instances of transcriptional readthrough, even if this may miss gene fusions occurring between adjacent genes - for example, as a result of tandem duplications or inversions [14]. Candidate fusion events between paralogous genes were excluded as likely mapping errors. Selecting gene-gene pairs supported by two or more short read pairs (Figure 1a) provided an initial list of 303 to 349 fusion candidates per cell line and 152 in normal breast. Of the initial 83 candidates tested, only seven (8.5%) were validated by RT-PCR, indicating that most of them represented false positives. We reasoned that if the process that gave rise to false positives involved PCR amplification or misalignment of short reads, we would expect that the artifactual reads spanning an exon-exon junction all align to the same position, whereas for a genuine fusion gene, we would expect a tiling pattern of short read alignment start positions across the fusion junction (Figure 1b). Examining the pattern among the initial list of fusion candidates indicated that all seven validated fusion genes displayed a tiling pattern. In contrast, the fusions we had been unable to validate had a frequently high number of identically mapping short reads (plus or minus a single base pair) aligning to the junction. These short reads also almost exclusively aligned to one of the exons. The paired-ends of identical short reads did not map within one to two bases of each other, suggesting misalignment, not PCR artifacts, is the likely reason for this phenomenon (data not shown). Utilizing the above-described criteria, we identified a total of 28 fusion gene candidates in the four breast cancer cell lines, whereas none were predicted in the normal breast sample.

### Fusion gene validation

**Figure 2:**
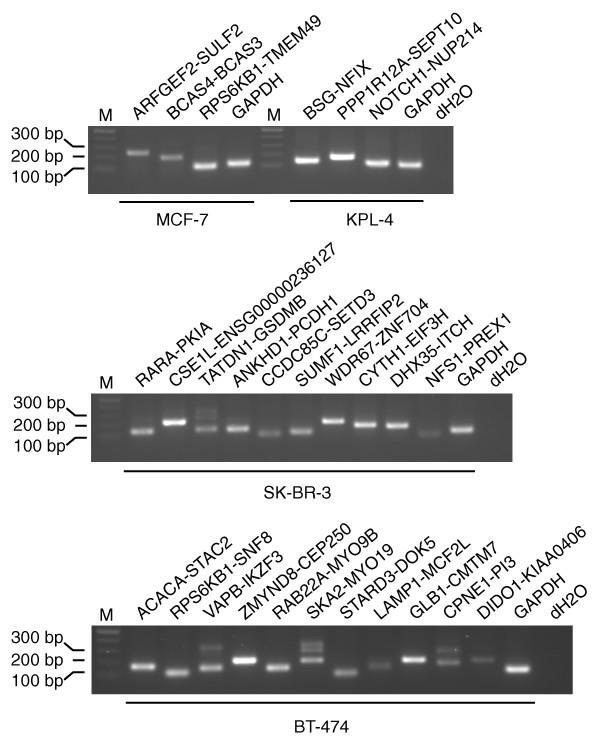
**Experimental validation of identified breast cancer fusion transcripts**. RT-PCR validation of fusions found in MCF-7 and KPL-4 (upper), SK-BR-3 (middle), and BT-474 (lower). Also shown is the marker and the negative control.

**Figure 3:**
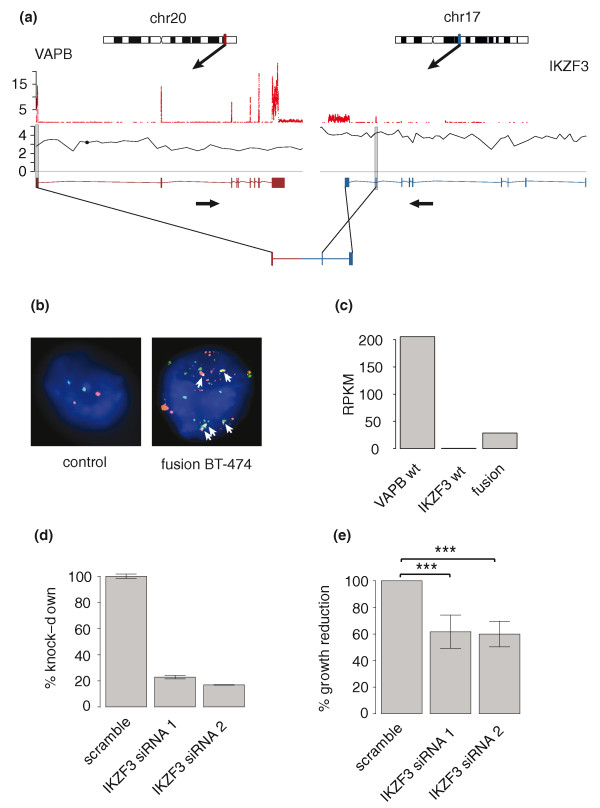
**Genomic structure, validation and functional significance of *VAPB*-*IKZF3***. **(a) **Exonic expression of *VAPB*-*IKZF3 *is indicated by sequencing coverage (red). Copy number changes measured by array comparative genomic hybridization (aCGH; black dots) in reference to normal copy number (horizontal grey line) and fusion break points (vertical grey line) are indicated. Gene structures are shown below the aCGH data. Arrows below gene structures indicate which strand the genes lie on. Fusion transcript structure is pictured below wild-type (wt) gene structures. **(b) **Interphase FISH showing amplification of *VAPB *and *IKZF3 *and the *VAPB*-*IKZF3 *fusion in BT-474. White arrows indicate gene fusions. **(c) **Expression of the 5' and 3' partner genes and the fusion gene. RPKM denotes reads per kilobase per million sequenced short reads. **(d) **Quantitative RT-PCR validation of small interfering RNA (siRNA) knock-down efficiency of cells transfected either with a scramble siRNA or with gene-specific siRNAs. Error bars show standard deviation. **(e) **CTG cell viability analysis of cells transfected either with a scramble siRNA or with gene-specific siRNAs. Asterisks indicate the statistical significance of growth reduction: ***P < 0.001. Error bars show standard deviation.

Using the improved bioinformatic pipeline described above, we were able to significantly reduce the number of false positive observations. We validated 27 of 28 (96%) fusion gene candidates using RT-PCR across the fusion break points followed by Sanger sequencing in the four breast cancer cell lines BT-474, KPL-4, MCF-7 and SK-BR-3 (Table 1, Figure 2). Of these, the three fusions identified in MCF-7 were previously known (*BCAS4-BCAS3*, *ARFGEF2-SULF2*, *RPS6KB1-TMEM49*), whereas all the others were novel. The validation of *NFS1*-*PREX1 *is tentative, as only a short segment of *NFS1 *was included in the fusion, complicating PCR primer design and subsequent sequencing. The fusion genes were unique to each cell line (Additional file 2). In order to ascertain whether the observed fusion mRNAs arise through rearrangements of the genomic DNA, we performed long-range genomic PCR (Additional file 3). Interphase FISH was also done to confirm selected fusions (Table 1, Figure 3b; Additional file 4). A genomic rearrangement was confirmed for 20 of 24 novel fusion genes. In the remaining cases, the lack of a PCR product may have been due to the difficulties with long DNA fragments in genomic PCR (Additional file 5), although we cannot exclude the possibility of mRNA *trans*-splicing in some of the cases [15].

**Table 1:** Identified and validated fusion gene candidates

Sample	5' gene	5' chromosome	3' gene	3' chromosome	Number of paired-end reads	Number of junction reads	In frame	Amplified	Genetic rearrangement validated
BT-474	*ACACA*	17	*STAC2*	17	57	72	Yes	Yes	Yes
BT-474	*RPS6KB1*	17	*SNF8*	17	43	68	Yes	Yes	Yes
BT-474	*VAPB*	20	*IKZF3*	17	41	26	Yes	Yes	Yes
BT-474	*ZMYND8*	20	*CEP250*	20	35	14	No	Yes	Yes
BT-474	*RAB22A*	20	*MYO9B*	19	9	12	No	Yes	Yes
BT-474	*SKA2*	17	*MYO19*	17	8	7	Yes	Yes	Yes
BT-474	*DIDO1*	20	*KIAA0406*	20	8	1	Yes	No	
BT-474	*STARD3*	17	*DOK5*	20	4	6	Yes	Yes	Yes
BT-474	*LAMP1*	13	*MCF2L*	13	5	3	No	No	Yes
BT-474	*GLB1*	3	*CMTM7*	3	6	2	Yes	No	Yes
BT-474	*CPNE1*	20	*PI3*	20	4	2	No	Yes	Yes
SK-BR-3	*TATDN1*	8	*GSDMB*	17	28	447	Yes	Yes	Yes
SK-BR-3	*CSE1L*	20	*ENSG00000236127*	20	10	20	Yes	Yes	
SK-BR-3	*RARA*	17	*PKIA*	8	13	10	Yes	Yes	Yes
SK-BR-3	*ANKHD1*	5	*PCDH1*	5	12	6	Yes	No	Yes
SK-BR-3	*CCDC85C*	14	*SETD3*	14	6	6	Yes	No	Yes
SK-BR-3	*SUMF1*	3	*LRRFIP2*	3	14	5	Yes	No	
SK-BR-3	*WDR67*	8	*ZNF704*	8	3	3	Yes	Yes	Yes
SK-BR-3	*CYTH1*	17	*EIF3H*	8	38	2	Yes	Yes	Yes
SK-BR-3	*DHX35*	20	*ITCH*	20	3	2	Yes	No	Yes
SK-BR-3	*NFS1*	20	*PREX1*	20	5	9	Yes	Yes	
KPL-4	*BSG*	19	*NFIX*	19	22	14	Yes	No	Yes
KPL-4	*PPP1R12A*	12	*SEPT10*	2	2	6	Yes	No	Yes
KPL-4	*NOTCH1*	9	*NUP214*	9	4	6	Yes	No	Yes
MCF-7	*BCAS4*	20	*BCAS3*	17	133	142	Yes	Yes	Previously reported
MCF-7	*ARFGEF2*	20	*SULF2*	20	17	25	Yes	Yes	Previously reported
MCF-7	*RPS6KB1*	17	*TMEM49*	17	2	7	Yes	Yes	Previously reported

A total of 24 novel fusion genes were identified in BT-474, SK-BR-3 and KPL-4. Three fusion genes detected in MCF-7 have been reported before and served as positive controls in our study. Two paired-end reads and two fusion junction spanning short reads were required for selecting a fusion candidate for further validation. In-frame prediction, copy number amplification (at least one of the fusion partner genes) and validation of the genomic rearrangement are indicated. Lower level copy number gains were excluded.

### Association with copy number breakpoints

**Figure 4:**
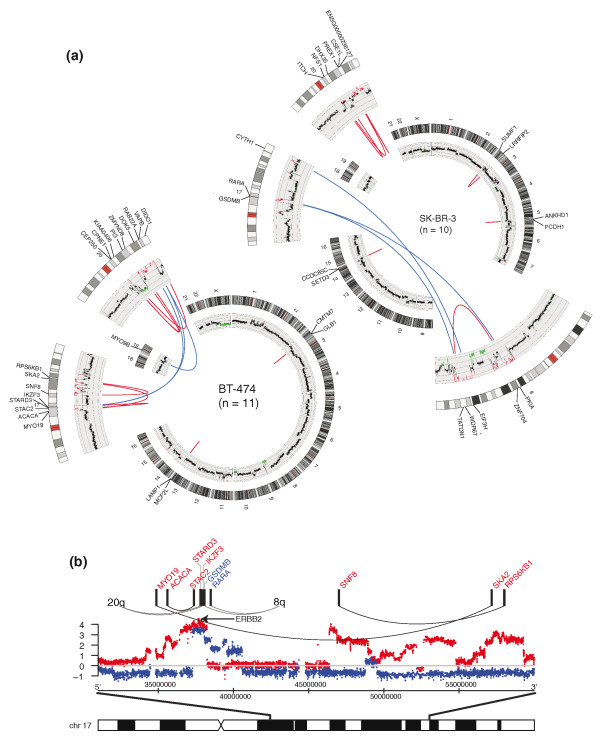
**Genomic rearrangements in SK-BR-3 and BT-474**. **(a) **Circos plots representing chromosomal translocations in SK-BR-3 (upper right) and BT-474 (lower left). Chromosomes are drawn to scale around the rim of the circle and data are plotted on these coordinates. Selected chromosomes involved in the fusion events are shown in higher magnification. Each intrachromosomal (red) and interchromosomal (blue) fusion is indicated by an arc. Copy number measured by aCGH is plotted in the inner circle where amplifications are shown in red and deletions in green. N denotes the number of fusion genes per cell line. **(b) **Fusion gene formation in the *ERBB2*-amplicon region. Fusion partner genes within and near the amplicon region are connected with black lines (both partners on chromosome 17), or location of the other partner is indicated (partner gene on different chromosomes). Smoothed aCGH profiles (log2) for SK-BR-3 (blue) and BT-474 (red) indicate copy number changes in reference to normal copy number (horizontal grey line). *ERBB2*, which is not fused (arrow), and chromosomal positions (bottom) are indicated.

Integration of RNA-seq with array comparative genomic hybridization (aCGH) data showed that, in 23 of 27 fusion genes, at least one partner gene was located at a copy number transition detected by aCGH, indicating that most of the fusion genes are not representing balanced translocations. In the case of 17 fusion genes, one or both genes were located at the borders of, or within, high-level amplifications on chromosomes 8, 17 and 20 (Figure 4a; Additional file 6). Since not all fusion genes in the proximity of amplicons were highly amplified, and many were not associated with DNA amplifications, we consider it likely that the association between fusion genes and DNA copy number changes is not markedly confounded by potential amplification-driven overexpression [16]. We also observed complex rearrangements, where multiple breaks in a narrow genomic region led to the formation of more than one gene fusion in the same sample. For instance, altogether six genes in the *ERBB2*-amplicons in BT-474 and SK-BR-3 took part in gene fusions (Figure 4b). As seen with the FISH analysis (Figure 3b; Additional file 4), the fusions were only seen in two to five copies per cell on average, indicating that the multiple genomic breakpoints required for the formation of high-level amplifications were probably contributing to the formation of the fusions as secondary genetic events.

Another important group of gene fusions was associated with breakpoints of low-level copy number changes, involving both gains and deletions. These are interesting in the sense that they represent the types of fusion events leading to gene activation with no association with gene amplifications. For example, this is the case for *TMPRSS2-ERG *and many leukemia-associated translocations [17]. Eight out of 27 fusion genes (*BSG-NFIX*, *CCDC85C-SETD3*, *DHX35-ITCH*, *CMTM7-GLB1*, *LAMP1-MCF2L*, *NOTCH1-NUP214, PPP1R12A-SEPT10 *and *SUMF1-LRRFIP2*) identified here were not associated with high-level gene amplifications, but typically had one of the fusion partners associated with a low-level copy number breakpoint, mostly gains or deletions. Interestingly, only the fusion gene *PPP1R12A-SEPT10 *in KPL-4 was not associated with either copy number transitions or changes at the location of either of the fusion counterparts as detected with the 1M probe aCGH.

### Structural properties of the novel fusion genes

**Figure 5:**
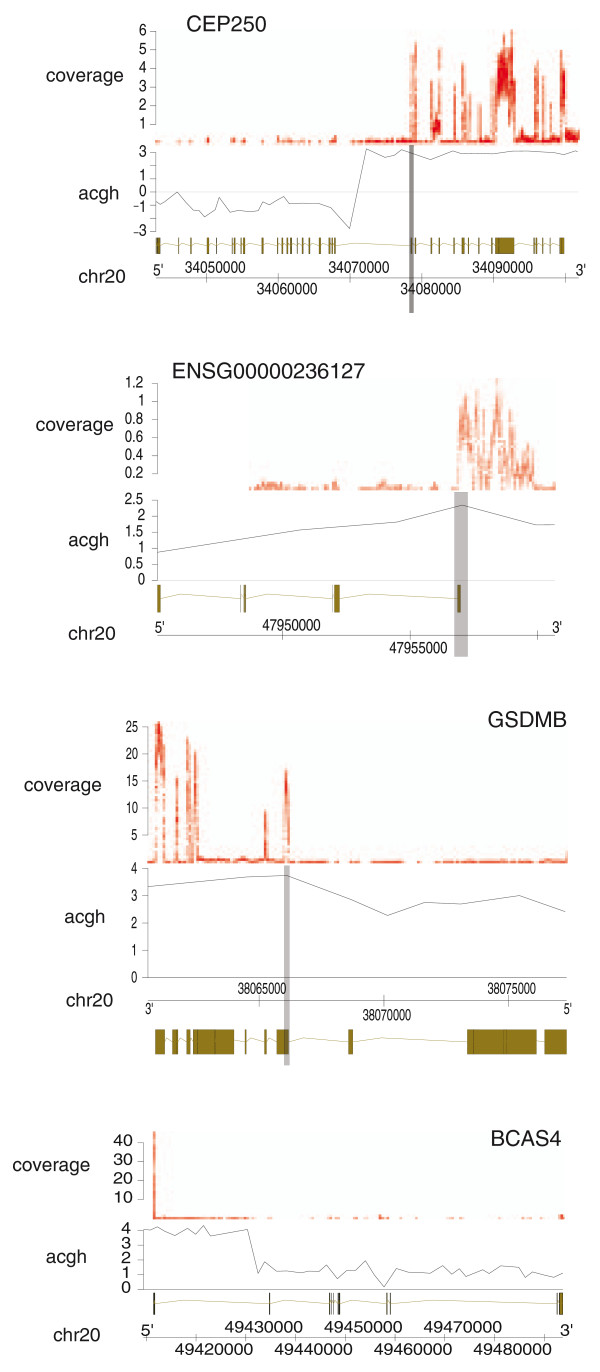
**Exclusive expression of the exons of the 3' partner genes taking part in the fusions**. Exonic expression of *CEP250 *in *ZMYND8*-*CEP250 *(upper), *ENSG00000236127 *in *CSE1L-ENSG00000236127 *(second from top), *GSDMB *in *TATDN1*-*GSDMB *(second from bottom) and *BCAS4 *in *BCAS3*-*BCAS4 *(lower) is indicated by sequencing coverage (red). Copy number changes measured by aCGH (black dots) in reference to normal copy number (horizontal grey line) and fusion break points (vertical grey line) are indicated. Chromosomal positions and transcript structures are shown below the aCGH data. Transcript structures above and below chromosome coordinates denote forward and reverse strand, respectively.

Several consistent patterns observed for the gene fusions suggest their potential importance. First, most of the fusions (23 of 27) were predicted to be in-frame (Table 1), assuming that the splicing pattern of the rest of the transcript is retained. Should the reading frame not be retained across the fusion junction, it would likely lead to appearance of a premature stop codon and the transcript would be degraded by nonsense-mediated mRNA decay. Therefore, it is possible that some of the highly expressed fusions that were predicted to be out-of-frame, such as *ZMYND8-CEP250*, may retain an intact open reading frame through alternative splicing or mutations that place the gene back in frame. Second, we observed 19 intra- and 8 interchromosomal translocations (Figure 4a; Additional file 6), which is in line with the previously observed pattern of intrachromosomal rearrangements occurring more frequently based on data from genomic sequencing [14]. Several (9 of 27) fusion partner genes were located on opposite strands, implying inversion, which in some cases has been followed by amplification of the rearranged region (for example, *ZMYND8-CEP250*). Third, the rearranged genes were occasionally exclusively expressed compared to their wild type partner genes (for example, *CEP250*, *IKZF3*, *GSDMB*, and *BCAS4*; Figure 5). Fourth, discovered fusions contributed both promoters (5' UTR; for example, *TATDN1-GSDMB*), coding sequences (for example, *ACACA-STAC2*) as well as 3' UTRs (for example, *CSE1L- ENSG00000236127*). Fifth, in the vast majority of the fusions (82%), at least one partner gene was located at a copy number breakpoint as revealed by aCGH, indicating that fusion gene formation is closely associated with unbalanced genomic rearrangements, particularly high-level amplifications [14,18]. Sixth, a number of fusion genes, such as *SKA2*-*MYO19 *and *CPNE1*-*PI3*, displayed alternative splicing at the fusion junction, suggesting fusion junction diversity (Figure 2).

### *VAPB-IKZF3 *fusion is required for the cancer cell phenotype

In order to gain insight into the functional role of the novel fusion genes, we performed small interfering RNA (siRNA) knock-down analysis targeting the parts of the 3' partner genes that are involved in the fusions. Based on the screen, the *VAPB-IKZF3 *fusion gene was selected for detailed validation. Knock-down of the IKAROS family zinc finger 3 (*IKZF3)*, which is part of the *VAPB-IKZF3 *fusion in BT-474, led to the inhibition of cancer cell growth. The *VAPB-IKZF3 *fusion gene is formed through a t(17;20)(q12;q13) translocation and consists of the promoter for *VAMP *(vesicle-associated membrane protein-associated protein B and C) and the carboxy-terminal part of *IKZF3*, which harbors two Zn-finger domains. *IKZF3 *was only detected as a fusion transcript, indicating activation of a quiescent gene by the fusion event (Figure 3a-c). Knock-down of *VAPB-IKZF3 *caused an 80% decrease in *VAPB-IKZF3 *expression (Figure 3d) and led to statistically significant (*P *< 0.001 for both siRNAs) cell growth inhibition in the BT-474 cells (Figure 3e). Two independent siRNAs targeting different regions of the fusion gene gave rise to the same phenotype. Thus, in the absence of detectable wild-type *IKZF3 *expression, the siRNA phenotype is reflecting the down-regulation of the fusion transcript (Figure 3d). This suggests that the growth of the BT-474 cells is dependent on the expression of *VAPB-IKZF3*.

## Discussion

In this study, we describe the identification of 27 fusion genes from breast cancer samples using paired-end RNA-seq combined with a novel bioinformatic strategy. This study therefore significantly increases the number of validated expressed fusion genes reported in breast cancer cells so far. This indicates the power of transcriptomic profiling by next-generation sequencing in that it can rapidly identify expressed fusion genes directly from cDNA, with a single lane of sequencing providing sufficient coverage. RNA-seq has been used before for fusion gene detection in a few solid tumor types [19-21]. However, in previous studies, fusion gene detection has been challenging because of the high rate of false positives [17,22]. Our sequencing procedure, coupled with an efficient bioinformatic pipeline, provides a cost-effective and highly specific platform for fusion gene detection in cancer, with a 95% success rate in validating the fusion transcripts.

mRNA *trans*-splicing has been reported to occur in human cells [15]. However, most of the fusion transcripts identified here can be attributed to underlying genetic alterations. In seven cases studied by FISH, a genomic fusion event was validated, while thirteen others were confirmed by genomic PCR, and the three fusions in MCF-7 cells were previously validated at the genomic level. The location of one of the fusion partners at a genomic copy number transition in 23 out of 27 cases also supports the conclusion that genomic alterations underlie the fusion transcripts in the vast majority of cases. This also suggests that the mechanism contributing to the fusion formation is linked to the underlying genomic DNA breaks. Fusions were associated with both low-level copy number gains and losses (9 of 27) as well as with high-level amplifications (17 of 27), especially within and between amplicons at 17q, 20q and 8q. For instance, we identified five different gene fusion events in which one or both partner genes are located in the *ERBB2*-amplicon at 17q12 in the BT-474 and SK-BR-3 cells (Figure 4b). Previous results have highlighted the fact that DNA level gene fusions often arise within high-level amplifications [23,24] but that a majority of them are not expressed [14]. The detailed characterization of the fusion gene events found here suggests that this may not always be the case.

The in-frame fusion genes found in the breast cancer cells included mostly fusions between protein coding regions (15 of 27) and promoter translocation events (8 of 27). The promoter translocations may fundamentally change the regulation of the genes, and link different oncogenic pathways. For example, promoter donating genes of interest in this regard include *RARA *and *NOTCH1*. Besides these two types of fusion, we also observed two cases of fusions of protein coding regions of the 5' partner primarily to the 3' UTR of the 3' gene (*CSE1L-ENSG00000236127 *and *ANKHD1-PCDH1*). These are predicted to encode truncated versions of the 5' proteins, with a new 3' UTR that could result in altered microRNA-mediated regulation of the gene.

Taken together, there are several lines of evidence from this study suggesting that the fusion genes may be functionally relevant. First, some fusions were clearly expressed higher than either or both of the wild-type genes, suggesting that the fusion event was linked to the deregulation and overexpression of the gene, and may have been selected for. For example, the *VAPB-IKZF3 *and *ZMYND8-CEP250 *fusion genes were expressed at significantly higher levels than their 3' partner genes (Figure 3c, Figure 5).

Second, we identified fusions involving genes taking part in oncogenic fusions in other cancers. *ACACA*, *RARA*, *NOTCH1 *and *NUP214 *are known to form translocations in various types of hematological malignancies while many other fusion genes involve suspected oncogenes, such as *RPS6KB1 *(*RPS6KB1-TMEM49 *and *RPS6KB1-SNF8*) [25], *GSDMB *(*TATDN1-GSDMB*) [26] and *MCF2L *(*LAMP1-MCF2L*) [27].

Third, a number of partners in gene fusions we reported here have previously been observed in other studies. For example, a *NUP214-XKR3 *translocation has been reported in leukemia cell line K562 [21]. *CYTH1 *was found translocated to *EIF3H *in our study, while Stephens *et al*. [14] identified the fusion *CYTH1-PRSAP1 *in breast cancer cell line HCC1599. *ANKHD1 *was in our study translocated to *PCDH1*, while Berger *et al*. [20] reported its fusion to *C5orf32 *in a melanoma short term culture.

Fourth, the knock-down studies by RNAi provided evidence of a functional role for *VAPB-IKZF3*, a fusion gene formed in conjunction with the 20q13 (*VAPB*) and the 17q12 amplicons (*IKZF3*). The fusion between *VAPB *and the hematopoietic transcription factor *IKZF3 *results in exclusive 'ectopic' expression of *IKZF3 *as a fusion transcript under the *VAPB *promoter. The decreased cell proliferation upon down-regulation of the *VAPB-IKZF3 *fusion gene in BT-474 cells suggests that this gene is necessary for the cancer cell growth and survival. *VAPB *has previously been proposed to function as an oncogene [28] while IKZF3 has been reported to interact with Bcl-xL, and Ras in T-cells, resulting in the inhibition of apoptosis [29,30]. *IKZF3 *is located at the most common telomeric breakpoint of the *ERBB2*-amplicon [31]. Interestingly, our preliminary analysis of clinical breast cancers shows that *IKZF3 *is overexpressed in a small subset of both HER2-positive as well as HER2-negative cancers, suggesting its expression may be elevated independent of *ERBB2 *amplification [32] (Additional file 7).

## Conclusions

Here, we present a large number of previously unknown gene fusions in breast cancer cells, whose identification was facilitated by the development of an improved bioinformatic procedure for detecting gene fusions from RNA-seq data. Our approach resulted in approximately 95% accuracy in classifying true fusion transcripts from raw RNA-seq data. These data indicate how gene fusions are much more prevalent in epithelial cancers than previously recognized and how they are often associated with copy number breakpoints. Therefore, sometimes deletions taking place in cancer may not be selected for due to an inactivation of a tumor suppressor gene in the region affected, but due to the generation of fusion genes at the breakpoints [3]. Similarly, fusion gene formation at the boundaries of the amplicons in cancer may modify or enhance the oncogenic impact caused by the increased copy number as demonstrated here for the potential functional importance of the *VAPB-IKZF3 *fusion gene. We present multiple lines of evidence suggesting the potential functional importance of the fusion genes, including the involvement of known oncogenic partner genes, exclusive expression of the partner genes as a fusion gene and RNAi-mediated knock-down studies. Finally, even if some of the fusion genes are not functionally critical or driver mutations, their detection from clinical specimens by RNA-seq at the cDNA level provides an attractive method to generate tumor-specific individual biomarkers for DNA based monitoring of cancer burden from patients' plasma [33,34].

## Materials and methods

### Cell culture

BT-474, MCF-7, and SK-BR-3 cells were obtained from American Type Culture Collection. KPL-4 was a kind gift from Dr Junichi Kurebayashi, Department of Breast and Thyroid Surgery, Kawasaki Medical School, Japan. MCF-7, KPL-4 and BT-474 cells were maintained in DMEM (Gibco, Invitrogen, NY, USA) supplemented with 10% fetal bovine serum (Source BioScience, LifeSciences, Nottingham, UK), 2 mM (MCF-7, KPL-4) or 4 mM (BT-474) L-glutamine (Gibco) and penicillin/streptomycin (Gibco). BT-474 cells were further supplemented with 1 mM sodium pyruvate and 0.01 mg/ml bovine insulin (Gibco). SK-BR-3 cells were maintained in McCoy's 5A medium (Sigma-Aldrich, St. Louis, MO, USA) with 10% fetal calf serum, 1.5 mM L-glutamine and penicillin/streptomycin. All cells were cultured at 37°C under 5% CO_2_.

### Sequencing library construction and paired-end RNA-

Total RNA from breast cancer cell lines (see above) was isolated using TRIzol (Invitrogen, Carlsbad, CA, USA) and subsequent phenol/chloroform extraction. The FirstChoice human breast total RNA was purchased from Applied Biosystems (Foster City, CA, USA). Messenger RNA templates were then isolated with oligo-dT Dynabeads (Invitrogen) according to the manufacturer's instructions and fragmented to average fragment size of 200 nucleotides by incubation in fragmentation buffer (Ambion, Austin, TX, USA) for 2 minutes at 70°C. We then used 1 μg of the resulting mRNA in a first strand cDNA synthesis reaction using random hexamer priming and Superscript II following the manufacturer's instructions (Invitrogen). To synthesize double-stranded cDNA, DNA/RNA templates were incubated with second strand buffer, dNTPs, RNaseH and DNA PolI (Invitrogen) at 16°C for 2.5 hours. cDNA was then purified (Qiagen PCR purification kit, Qiagen, Hilden, Germany). To ensure the proper fragment distribution pattern and to calculate template concentration, cDNA was analyzed using Bioanalyzer DNA 1000 kit (Agilent Technologies, Santa Clara, CA, USA). End repair of template 3' and 5' overhangs was performed using T4 DNA polymerase, Klenow DNA polymerase and T4 PNK (New England BioLabs, Beverly, MA, USA). Template and enzymes were allowed to react in the presence of dNTPs and ligase buffer supplemented with ATP (New England BioLabs) at 20°C for 30 minutes, purified (Qiagen PCR purification kit) and subjected to A-base addition through incubation at 37°C for 30 minutes with Klenow 3' to 5' exo-enzyme, Klenow buffer and dATP (New England BioLabs). Following purification with a Qiagen MinElute kit, paired-end adaptors were ligated onto the templates with Ultrapure DNA ligase (Enzymatics, Beverly, MA, USA) or quick DNA ligase (New England BioLabs) at 20°C for 15 minutes and purified as above. Ligation efficiency was assessed with PCR amplification. cDNA templates were then size selected through gel purification and paired-end libraries created using Pfx polymerase (Invitrogen) and subsequently purified and their concentration calculated. The median size of the MCF-7 and KPL-4 paired-end library was around 100 nucleotides, whereas for BT-474 and SK-BR-3, two library preparations were done, with median insert sizes of 100 and 200 nucleotides, respectively. For the normal breast, the median insert size of the sequencing library was 200 nucleotides. The paired-end sequencing was performed using the 1G Illumina Genome Analyzer 2X (Illumina) according to the manufacturer's instructions. The following primers were used (an asterisk denotes phosphorothiate modification): adaptor ligation, SLX_PE_Adapter1_ds 5'[Phos]GATCGGAAGAGCGGTTCAGCAGGAATGCCGA*G, SLX_PE_Adapter1_us 5'A*CACTCTTTCCCTACACGACGCTCTTCCGATCT; PCR library, SLX_PE_PCR_Primer1f 5'A*ATGATACGGCGACCACCGAGATCTACACTCTTTCCCTACACGACGCTCTTCCGATC*T, SLX_PE_PCR_Primer1r 5'C*AAGCAGAAGACGGCATACGAGATCGGTCTCGGCATTCCTGCTGAACCGCTCTTCCGATC*T.

The raw sequencing data have been deposited in the NCBI Sequence Read Archive [SRA:SRP003186].

### Sequence alignment

Ensembl versions 55 (BT-474, MCF-7, KPL-4 and normal breast) and 56 (SK-BR-3), both utilizing version NCBI37 of the human genome, were used for all short read alignments. Throughout the paper, Ensembl version 55 was used for all analyses relating to BT-474, MCF-7, KPL-4 and normal breast, whereas version 56 was used for SK-BR-3. Short reads obtained from Genome Analyzer II (Illumina) (FASTQ files: s_*_*_sequence.txt) were trimmed from 56 bp to 50 bp. Short reads aligning to human ribosomal DNA (18S, 28S, 5S, 5.8S) and complete repeating unit ribosomal DNA were filtered out. Additionally, short reads mapping on contaminant sequences (for example, adaptor sequences) were filtered out. The remaining short reads were aligned against the human genome and the splice-site junction sequences of each gene (here a splice-site junction sequence is the sequence on the transcript level where two consecutive exons are joined). The mapped short reads were divided into three categories: short reads that do not align in the genome; short reads that align uniquely; and short reads that align to multiple loci in the genome and splice-site junction sequences for each gene. For alignment a maximum of three mismatches are allowed and Bowtie software version 0.11.3 [35] was used for short reads alignment. Short reads that aligned uniquely and short reads that did not align were compared again against all Ensembl transcripts. Here the paired-end reads were used to find the fusion gene candidates, that is, paired-end reads that map on two transcripts from different genes.

### Fusion gene identification

Uniquely aligning short reads were assigned to genes based on the transcript of the gene to which they aligned. A preliminary set of fusion genes was identified by selecting all the gene-gene pairs for which there were at least two (MCF-7, KPL-4, normal breast) or three (BT-474, SK-BR-3) short read pairs such that one end aligns to one of the genes and the other to the other. A higher threshold for BT-474 and SK-BR-3 was used to account for greater sequencing depth in these cell lines and keep the proportion of false positive findings constant from sample to sample. Paralogous gene-gene pairs were identified based on paralog status in Ensembl. Gene biotype was also obtained from Ensembl. Two genes were defined as non-adjacent if there was a third gene, of any biotype, such that both its start and stop positions lie between the two other genes. To identify the exon-exon fusion junction, a database of artificial splice-site junctions was built by generating all the potential exon-exon combinations between gene A-gene B and B-A for each pair of candidate-fusion genes. Short reads that did not align on either the genome or the transcriptome were aligned against the junction database in order to locate the exact fusion point, that is, between which exons the gene fusion takes place. Junctions spanning short reads were required to align at least 10 bp to one exon. This step also defines which gene is the 5' fusion partner. A minimum of two junction-spanning short reads were required. The initial set of 83 candidates were selected based on the number of paired-end and junction spanning reads as well as each gene taking part in only a few fusions per sample. The final 28 fusion gene candidates were prioritized for laboratory validation based primarily on the number and position of unique short read alignment start positions across the fusion junction (Figure 1) and secondarily on location at a copy number transition. One million oligo Agilent aCGH data were combined with sequencing data by drawing images of sequencing coverage and copy number data along with the structure of each candidate gene. Parsing of alignments and other custom analyses were done with in-house developed Python tools. Fusion gene prioritization was done using custom tools built using R [36] and Bioconductor [37].

### Fusion gene characterization

Fusion gene frame was predicted by creating all possible fusions between those Ensembl transcripts of both genes that contain the fused exons. A fusion transcript is predicted to be in-frame if any of the transcript-transcript fusions, or their potential splice variants, retain the same frame across the fusion junction. Expression of fusion genes and wild-type parts of the fused genes was calculated as uniquely mapped reads per kilobase of gene sequence per million mapped reads (RPKM). Fusion gene expression was calculated from the number of short reads aligning to the fusion junction. To determine if any of the fused genes has previously been reported to take part in translocations, all 5' and 3' genes were compared against the Mitelman Database of Chromosome Aberrations [38]. To determine if fused genes have otherwise been mutated in cancer, all 5' and 3' genes were compared against the COSMIC database version 45 [39] and the Cancer gene census [40]. Coverage for each of the fused genes was determined by calculating how many times each nucleotide of the gene was sequenced. Coverage plots were drawn using R [36] and the GenomeGraphs [41] package in Bioconductor [37]. Plots illustrating the discovered fusions and their association to copy number changes were drawn using the Circos software [42].

### aCGH

aCGH was performed as described previously [43] following the protocol provided by Agilent Technologies (version 6), including minor modifications. Briefly, genomic DNA was extracted using TRIzol (Invitrogen) and purified by chloroform extraction and subsequent ethanol precipitation. Three micrograms of digested sample or reference DNA (female genomic DNA; Promega, Madison, WI, USA) was labeled with Cy5-dUTP and Cy3-dUTP, respectively, using Genomic DNA Enzymatic Labeling Kit and hybridized onto SurePrint G3 Human 1M oligo CGH Microarrays (Agilent). To process the data a laser confocal scanner and Feature Extraction software (Agilent) were used according to the manufacturer's instructions. Data were analyzed with DNA Analytics software, version 4 (Agilent). Raw aCGH data have been deposited in Gene Expression Omnibus [GEO:GSE23949].

### RT-PCR and quantitative RT-PCR

The predicted fusion genes were validated by RT-PCR followed by Sanger sequencing. Fusion junction sequences are listed in Additional file 8. For the RT-PCR reactions 3 μg of total RNA was converted to first-stranded cDNA with random hexamer primers using the High-Capacity cDNA Reverse Transcription kit (Applied Biosystems) according to the manufacturer's instructions. RT-PCR products were gel-purified (GE Healthcare, Little Chalfont, UK) and cloned into pCRII-TOPO cloning vector (Invitrogen). All clones were confirmed by sequencing using an ABI Prism 3730×l DNA Sequencer (Applied Biosystems). Quantitative RT-PCR reactions were carried out on a LightCycler^®^480 (Roche Applied Science, Penzberg, Germany) using DyNAmo SYBRGreen PCR kit (Finnzymes, Espoo, Finland). Primers specific either for wild-type partner genes or fusion genes were used in RT-PCR and quantitative RT-PCR and are listed in Additional file 9. *GAPDH *was used as internal reference gene. All experiments were performed in triplicates.

### Long-range genomic PCR

Genomic DNA was isolated and purified as described above. Genomic DNA amplifications were performed using Expand Long Range dNTP pack kit (Roche) according to the manufacturer's instructions. For each fusion gene pair, the maximum size of intervening intronic sequence was calculated and primers (Additional file 5) were placed such that the amplicon encompassed the fusion junction. Primer sequences are listed in Additional file 9.

### FISH

Interphase FISH was performed for selected fusion genes. The BAC probes were selected to lie as close as possible on each side of the breakpoint. The BAC clones were obtained from ImaGenes (Berlin, Germany) and grown overnight in LB media supplemented with chloramphenicol and DNA was isolated with Qiagen Plasmid Maxi-kit. The probes flanking the breakpoint were labeled differentially with green-dUTP (Abbott, Des Plaines, IL, USA) and orange-dUTP (Abbott). Twenty nanograms of each probe were used per hybridization. Denaturation of probe and target DNA was performed for 5 minutes at 87°C, followed by hybridization in a humidity chamber overnight at 47°C. The cover glasses were then removed, and the slides were washed twice in 4× SSPE for 10 minutes at 37°C and 47°C, and the slides were dehydrated in graded alcohol, 10 minutes in hexanol:isopropanol, 5 minutes in isopropanol before rehydration in graded alcohol and 5 minutes in 0.1× phosphate-buffered saline. The slides were mounted with antifade mounting medium containing 4',6'-diamino-2-phenylindole (Vectashield^® ^DAPI, Vector Laboratories, Burlingame, CA, USA) as a counterstain for the nuclei. Evaluation of fluorescence signals was carried out in an epifluorescence microscope. Selected cells were photographed in a Zeiss Axioplan 2 microscope equipped with an Axio Cam MRM CCD camera. Image analysis was performed using Axio Vision software. FISH probe identifiers are listed in Additional file 10.

### siRNA knock-down experiments

The double-stranded siRNA oligonucleotides were obtained from Qiagen and Applied Biosystems. Plates pre-printed with 4 μl of siRNA stock solutions were diluted with 10 μl of transfection reagent-Opti-MEM (Invitrogen), and an appropriate amount of cells (1,500 to 2,000 per well) were plated in 35 μl of media. KPL-4, SK-BR-3 and BT-474 cells were reverse transfected using Silentfect (Bio-Rad, Hercules, CA, USA), Hiperfect (Qiagen) and Dharmafect 1 (Thermo Scientific, Lafayette, CO, USA), respectively. AllStars Negative Control (Qiagen), AllStars cell death control (Qiagen) and siRNA against PLK1 and KIF11 were used as controls in all experiments. The final siRNA concentration was 13 nmol/L. For proliferation assays the total cell number was assayed using the CellTiter-Glo Cell Viability Assay (Promega) 72 hours after the transfection according to the manufacturer's instructions. The EnVision Multilabel Plate Reader (Perkin-Elmer, Waltham, MA, USA) was used for signal quantification. Screening data were normalized in a plate-wise manner using the B-score method [44]. All gene-targeting siRNAs were screened in three replicate wells per plate and screens were repeated three times. For hit identification, all B-score values for an siRNA against a specific cell line were treated as a group and compared to the AllStars negative control siRNAs from the corresponding cell line using the Mann-Whitney U-test. Bonferroni correction was used to correct for multiple testing. siRNAs with a *P*-value < 0.05 were considered hits.

To validate the hits from the BT-474 screens, cells were reverse transfected as above with IKZF3 siRNA 1 (Hs_IKZF3_3, Qiagen) and IKZF3 siRNA 2 (Hs_ZNFN1A3_5, Qiagen) in 96-well plates using siRNAs and 10,000 cells per well. CellTiter-Glo Cell Viability Assay (Promega) was used as an endpoint measure after 168 hours.

## Abbreviations

aCGH: array comparative genomic hybridization; BAC: bacterial artificial chromosome; bp: base pair; DNA-seq: DNA-sequencing; FISH: fluorescence *in situ *hybridization; RNAi: RNA interference; RNA-seq: RNA-sequencing; siRNA: small interfering RNA; UTR: untranslated region.

## Competing interests

The authors declare that they have no competing interests.

## Authors' contributions

HE designed the study, and contributed the majority of data analysis and writing of the manuscript. AM designed the study, and contributed the sequencing and validation experiments as well as to manuscript writing. SK designed the study, and contributed the sequencing and validation experiments as well as to manuscript writing. DN contributed sequence alignment. VH contributed siRNA screening and Protein Lysate MicroArray analysis. KK contributed to data validation. IHR contributed FISH analysis. SN contributed siRNA screening. MW contributed aCGH experiments. ALBD supervised FISH analysis and data validation. OK supervised the entire project and participated in manuscript writing and editing.

## Supplementary Material

Additional file 1 **Table showing paired-end RNA-seq summary statistics**. Click here for file

Additional file 2 **Cell line specificity of the novel fusion genes**. RT-PCR validation of fusion genes discovered in BT-474 (left), SK-BR-3 (middle) and KPL-4 (right) with a panel of breast cancer cell lines and normal breast tissue. *GAPDH *was used as the internal reference gene. Click here for file

Additional file 3 **Long-range genomic PCR on fusion genes**. Genomic PCR across the fusion junctions of selected gene fusions in BT-474 (left), KPL-4 (middle) and SK-BR-3. Click here for file

Additional file 4 **FISH analysis of fusion genes. (a) **Interphase FISH showing amplification of *STARD3 *and *DOK5 *(left), *VAPB *and *CYTH1 *(middle) and *RPS6KB1 *and *SNF8 *(right) in BT-474 cells but not in control cells. White arrows indicate fused genes. Coloring of the gene names coincides with labeling of the BAC clones used. **(b) **Interphase FISH showing amplified signals of *BSG *and *NFIX *(left) and *NOTCH1 *and *NUP214 *(right) in KPL-4. Normal copy number of both genes is present in control cells. **(c) **Interphase FISH analysis showing many copies of *CYTH1 *and *EIF3H *(left) and *TATDN1 *and *GSDMB *(right) in SK-BR-3. In contrast, only normal copy number of these genes is visible in control cells. Click here for file

Additional file 5 **Combined maximum intron sizes**. Click here for file

Additional file 6 **Genomic rearrangements in KPL-4 and MCF-7**. Circos plots representing chromosomal translocations in KPL-4 (bottom) and MCF-7 (top). Chromosomes are drawn to scale around the rim of the circle and data are plotted on these coordinates. Selected chromosomes involved in the fusion events are shown in higher magnification. Each intrachromosomal (red) and interchromosomal (blue) fusion is indicated by an arc. Copy number measured by aCGH is plotted in the inner circle where amplifications are shown in red and deletions in green. N denotes the number of fusion genes per cell line. Click here for file

Additional file 7 **Expression of *IKZF3 *and *ERBB2 *in breast cancer**. Genesapiens.org plot showing a scatterplot comparing *IKZF3 *and *ERBB2 *expression in a set of 761 breast tumors profiled on Affymetrix gene expression microarrays. Click here for file

Additional file 8 **Fusion junction sequences**. Click here for file

Additional file 9 **Primer sequences used in the study**. Click here for file

Additional file 10 **FISH probes used for validation**. Click here for file
