# Supplementary material for: Identification of fusion genes in breast cancer by paired-end RNA-sequencing
Source: Genome Biol. 2011 Jan 19;12(1):R6. doi: 10.1186/gb-2011-12-1-r6 (PMC3091304; doi:10.1186/gb-2011-12-1-r6)
Supplement: Additional file 1 — Table showing paired-end RNA-seq summary statistics. [file gb-2011-12-1-r6-S1.PDF]

Additional file 5. Paired-end RNA-seq summary statistics

| | MCF-7 | BT-474 | SK-BR-3 | KPL-4 | Normal breast |
|---|---|---|---|---|---|
| Number of lanes | 3 | 2 | 2 | 1 | 1 |
| Unique reads | 9.391.444 | 22.729.557 | 32.131.811 | 7.979.513 | 8.554.829 |
| Multiple reads | 1.551.671 | 3.053.108 | 4.832.155 | 1.031.888 | 1.261.876 |
| Non-mapping reads | 1.862.559 | 3.947.238 | 5.373.572 | 1.188.192 | 1.317.916 |
| Total | 12.805.674 | 29.729.903 | 42.337.538 | 10.199.593 | 11.134.621 |
