# Supplementary figures and images for: Identification of fusion genes in breast cancer by paired-end RNA-sequencing
Source: Genome Biol. 2011 Jan 19;12(1):R6. doi: 10.1186/gb-2011-12-1-r6 (PMC3091304; doi:10.1186/gb-2011-12-1-r6)

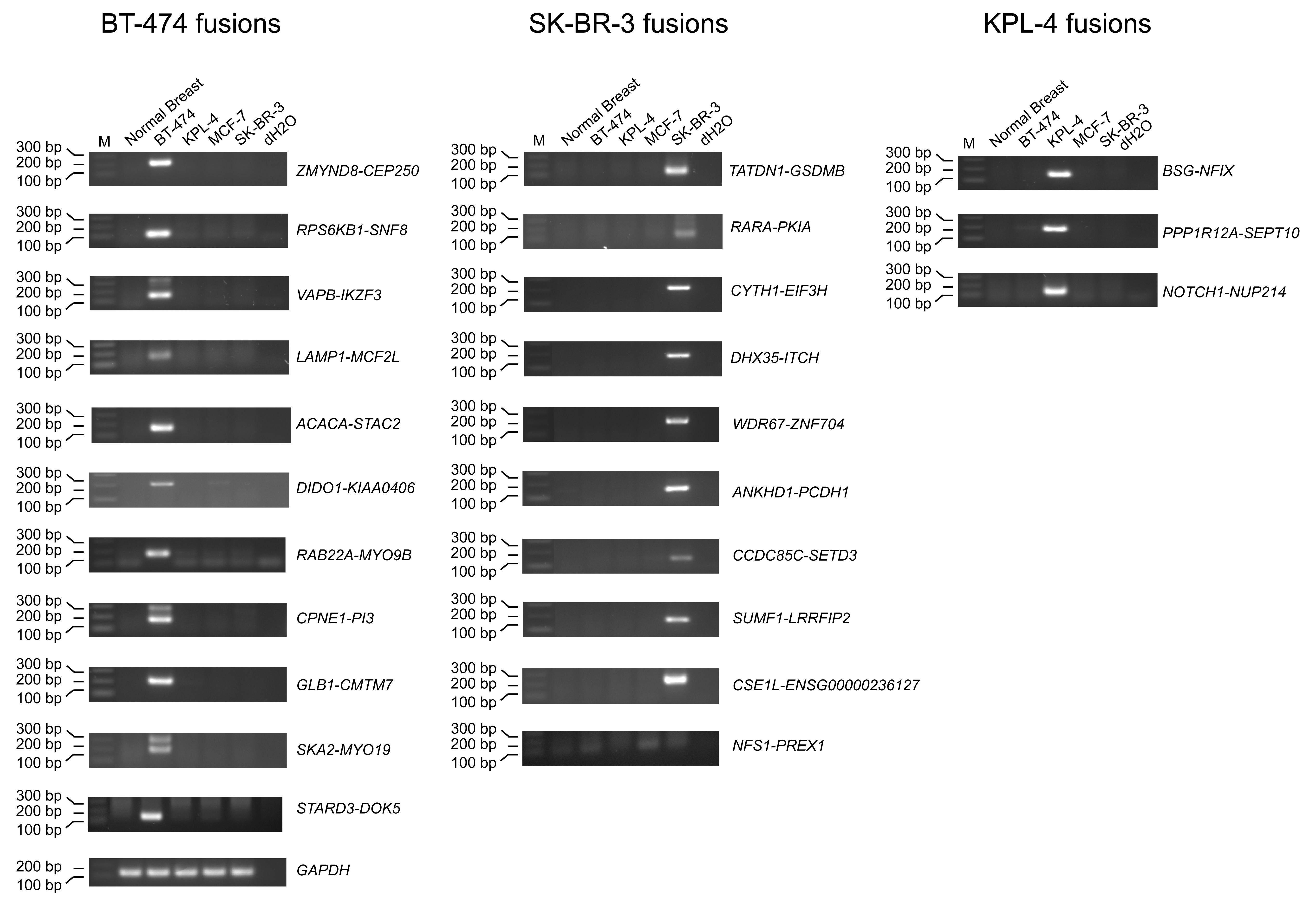

Supplement: Additional file 2 — Cell line specificity of the novel fusion genes. RT-PCR validation of fusion genes discovered in BT-474 (left), SK-BR-3 (middle) and KPL-4 (right) with a panel of breast cancer cell lines and normal breast tissue. GAPDH was used as the internal reference gene. [file gb-2011-12-1-r6-S2.TIFF]

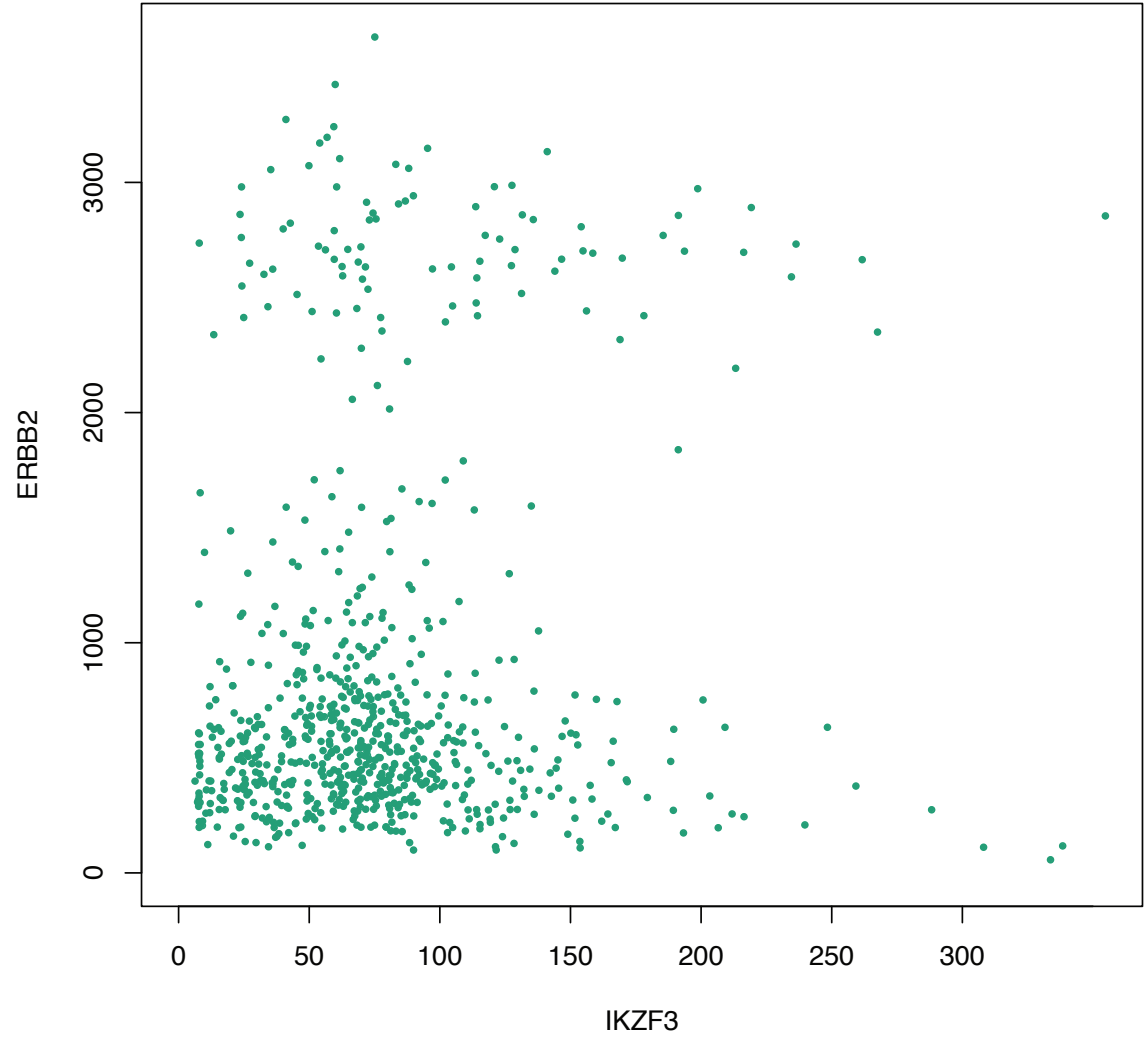

Supplement: Additional file 7 — Expression of IKZF3 and ERBB2 in breast cancer. Genesapiens.org plot showing a scatterplot comparing IKZF3 and ERBB2 expression in a set of 761 breast tumors profiled on Affymetrix gene expression microarrays. [file gb-2011-12-1-r6-S7.PDF]
