# Supplementary material for: Identification of fusion genes in breast cancer by paired-end RNA-sequencing
Source: Genome Biol. 2011 Jan 19;12(1):R6. doi: 10.1186/gb-2011-12-1-r6 (PMC3091304; doi:10.1186/gb-2011-12-1-r6)
Supplement: Additional file 5 — Combined maximum intron sizes. [file gb-2011-12-1-r6-S5.PDF]

Sample	Fusion gene	Sum of intron sizes	Genetic rearrangement validated
BT474	ACACA-STAC2	8279	yes
BT474	RPS6KB1-SNF8	17941	yes
BT474	VAPB-IKZF3	39183	yes
BT474	ZMYND8-CEP250	13336	yes
BT474	RAB22A-MYO9B	75434	yes
BT474	SKA2-MYO19	24900	yes
BT474	DIDO1-KIAA0406	28506	
BT474	STARD3-DOK5	49183	yes
BT474	LAMP1-MCF2L	12580	yes
BT474	GLB1-CMTM7	66485	yes
BT474	CPNE1-PI3	23137	yes
SKBR3	TATDN1-GSDMB	18423	yes
SKBR3	CSE1L-ENSG00000236127	4737	
SKBR3	RARA-PKIA	77912	yes
SKBR3	ANKHD1-PCDH1	6050	yes
SKBR3	CCDC85C-SETD3	58118	yes
SKBR3	SUMF1-LRRFIP2	33819	
SKBR3	WDR67-ZNF704	62205	yes
SKBR3	CYTH1-EIF3H	72623	yes
SKBR3	DHX35-ITCH	9388	yes
SKBR3	NFS1-PREX1	904	
KPL4	BSG-NFIX	872	yes
KPL4	PPP1R12A-SEPT10	14595	yes
KPL4	NOTCH1-NUP214	28922	yes
MCF7	BCAS4-BCAS3	261799	previously reported
MCF7	ARFGEF2-SULF2	39434	previously reported
MCF7	RPS6KB1-TMEM49	13082	previously reported
