# Supplementary material for: Identification of fusion genes in breast cancer by paired-end RNA-sequencing
Source: Genome Biol. 2011 Jan 19;12(1):R6. doi: 10.1186/gb-2011-12-1-r6 (PMC3091304; doi:10.1186/gb-2011-12-1-r6)
Supplement: Additional file 8 — Fusion junction sequences. [file gb-2011-12-1-r6-S8.PDF]

Fusion gene junction sequences:
Fusion junction indicated by an asterisk

BSG-NFIX
TGCTGGTCTGCAAGTCAGAGTCCGTGCCACCTGTCACTGACTGGGCCTGGTACAAGATCACTGACTCTGAG
GACAAG*GATGAGTTCCACCCGTTCATCGAGGCACTGCTGCCTCACGTCCGCGCTTTCTCCTACACCTGGTT
CAACCTGCA

PPP1R12A-SEPT10
CAAAACCCCTGGCTTCTGTAACTAATGCCAACACTTCTAGTACACAAGCAGCTCCTGTAGCTGTTACAACAC
CTACTGTGTCATCAGGTCAAGCAACACCTACATCACCTATTAAAAAG*AAAAGAGAAAACATTCGTTCGTTGA
CTATGTCTGGCCATGTTGGTTTTGAGAGTTTGCCTGATCAGCTGGT

NOTCH1-NUP214
GGTGAGACCTGCCTGAATGGCGGGAAGTGTGAAGCGGCCAATGGCACGGAGGCCTGCGY*TTCTTCAGTG
CCCTACTCCACAGCCAAAACACCTCACCCAGTGTTGACCCCAGTGGCTGCTAACCAAGCCAAGCA

DIDO1-KIAA0406
GTTGGCGCCAGAGTCAAAAGGCGTCGGCCCTCTGGCAAGATGGCTGCTGCGGAGGCGTTGGAGCGCGGA
AATCTGGAACCGGGATGGCGACGTCTACACTGAGTCGGAGGCGAAG*CCCAGTGGTTCCCAGACACAGGTA
ATCTTGGGCACCTCCAAGAGCAAAGTTTAGGAGAAGAGGGAAGTCATTTGAACCAAAGACCAGCRGCTC

RPS6KB1-SNF8
GAGGACATGGCAGGAGTGTTTGACATAGACCTGGACCAGCCAGAGGACGCGGGCTCTGAGGATGAGCTGG
AGGAGGGG*GCCAAGTATAAGGAGCGAGGGACGGTCTTGGCTGAGGACCAGCTAG

VAPB-IKZF3
CTAAGGAACATGGCGAAGGTGGAGCAGGTCCTGAGCCTCGAGCCGCAGCACGAGCTCAAATTCCGAG*CAA
GTGCGGAGGCAAGACACATCAAAGCAGAGATGGGAAGTGAAAGAGCTCTCGTACTGGACAGATTAGCAAGC
AATGTGGCAAAACGAAAAAGCTCAA

ACACA-STAC2
TTGCTGTAGAAACCCGAACAGTAGAACTAAGTATCCCAGCTGATCCAGCAAACCTGGATTCTGAAGCCAAG*
CTCCAGCGATTCAAGCGCTCCCTCTCCCTCAAGACCATCCTCCGAAGTAAGAGCTTGGAGAACTTCTTCCTT
CGCTCGG

ZMYND8-CEP250
GCCATCTTTTACTGCTGTTGGAACACCAGCTACTGTGACTACCCCTGCCAGCAAGCCCACTGGCCTGAGCA
CATGAAGTCCTGCACCCAGTCAG*GAGAAGGAGCGCTCCTGGCACCAGTAGGAGCTGGCAAAGGCTCTGGA
GAGCTTAGAAAGGGAAAAAATGGAGCTGGAAATGAGGCTAAAGGAGCAGCAGACAGAAATGGAGGCCAT

RAB22A-MYO9B
GTATTGTGTGGCGGTTTGTGGAAGACAGTTTTGATCCAAACATCAACCAACAATAGG*GCTTTTGGAAATGCC
AAGACAGCCCACAACAACAACTCCAGCCGGTTTGGGAAATTCATCCAAGTCAGCTACCTAGAGAGTGGCAT
CGTGAGAGG

SKA2-MYO19
GCGAGATGTTGAGTGACAGCTGTCCAATGGAGGCCCTCCGTGCTAGTACGGCTTGCGGCTTAAGCGCCGC
CGCGGTCTGCGGAATGTCAACTATTCAACATGGAGGCGGAGGTCGATAAGCTGGAACTGATG*GCCTGCTG
GATGTGTATGGATTTGAATCATTTCCTGACAACAGTCTGGAACAGTTGTGCATCAACTACGCCAATGAG

STARD3-DOK5
GTGGCTGACATGGAGCAG*CTCCAGATGAAGATGAGTGAGCGGGCCGCCTCGCTGAGCACCATGGTGCCC
CTGCCTCGCAGCGCCTACTGGCAGCACATCACA

LAMP1-MCF2L

GGTAACGCCGCTGTCTCTAACGCCAGCCCTTGGCGCCCGCGCCCCGCCACCGCAGCGCCCGGCGACCCC
TGCTGCTGCTACTGCTGTTGCTGCTGCTCG*GTCATAATGCTGAGCTCCGTACCAGACTTACACGGTTACAT
CGATAAGTCGCAGCTG

GLB1-CMTM7
CGGCCTTTTATATGGGGAACTTCTCCATTCCCAGTGGGATCCCAGACTTGCCCCAGGACACCTTTATCCAGT
TTCCTGGATGGACCAAG*GTCACCCTGCTGCTTGCCTTCATCTGTGTGCGGAGCTCCCTGTGGACCAACTAC
AGCGCCTACAGCTACTTTGAAGTGGTCACCATTTGCGACTTGATAATGATCCTCGCCTTTTACCTG

CPNE1-PI3
TCGCCACTTCTTCTCTGGATTGACTATTCCTGATGGGGGCGTGCATATTGTAGGGGGTGAACTGG*TTCTAG
AGGCTGGGAAGTCCAAGATCAAGGCACCAGCAGATTCAGCATCTGTTCCTGTTAAAGGTCAAGACACTGTCA
AAGGCCGTGTTCCATTCAATGGACAAGATCCCGTTAAAGGACAAGTTTCAGTTAAAGGTCAAGATAAAGTCA
AAGCGCAAGAGCCAGTCAAAGGTC

RARA-PKIA
TCCTGAATCGAGCTGAGAGGGCTTCCCCGGTTCTCCTGGGAACCCCATCGGCCCCCTGCCAGCACACACCT
GAGCAG*TTTCCTGACTTTCTGAGAAGCCCTGGTTTCCCCAAAGAAGTGATTTCTGATAGAAATCTGAAGGTC
ATCTCCAA

TATDN1-GSDMB
GCAGTCATGAGTCGCTTCAAGTTTATCG*GAAGCTGAAGAGGGAACTACCCTTTTCATTCCGATCAATTAATA
CGAGAGAAAACCTGTATCTGGTGACAGAAACTCTGGAGACGGTAAAGGAGGAAACCCTGAAAAGCGACCGG
CAATATA

CSE1L-ENSG00000236127
TAATGCCGCACTCTATGCACAAAAGTACGATGAAGAATTCCAGCGATACCTGCCTCGTTTTGTTACAGCCAT
CTGGAATTTACTAGTTACAACGGGTCAAGAGGTTAAATATGATTTG*GAACAAGATGTGGCTGTGGCAAGCG
GCCGCCATCCCCGGTGCCGAGGGACCTGGGAGAAGCTAGGGCAGCTCCGGCATCGTTCCTGCCCTGTGGT
CCACAGCCTCGTCTTCAT

ANKHD1-PCDH1
CAGTGCAGGTCATGTGGAAGTTGCAAGAGTTCTTTTAGATCATGGTGCAGGCATCAACACTCATTCTAATGA
ATTCAAAGAAAGTGCTCTAACACTTGCTTGCTACAAAG*ACCTTCGCCCTTTGCCTGATGTCGCCATGACAGG
CACATGTACC

CCDC85C-SETD3
AAGGGTAGTTTGTTGGCATGAGGATGGGT*CTGTGCGAGGAGCTTGTCACCCTCCAGCAGCCTCACTTTGCT
CTCCAGTTGCCTGATGTAGGTGGATGAGGGA

SUMF1-LRRFIP2
CTTCTGGGAAAGACCGAGTGAAGAAAGGTGGATCCTACATGTGCCATAGG*GCAGAGGCAAGGCTGGCAGC
AAAACGGGCTGCCCGGGCAGAAGCAAGAGATATACGCATGAGAGAACTGGAACGA

WDR67-ZNF704
GTGCATGCATCAGGGAAATATGCCATCACAACTTCTTCTGATACAGCACAATTATGGGACTTGGATACCTTTC
AGAGAAAAAGAAAGCTGAATATTCGCCAGTCTGTGGGTATACAGAAG*GTGGGGAGCATTAAGCGGGAAATG
ACCTTCACATTTCAGTCAGAGGACTTAAAACGTGACTGTGGTAAAAAAATGTCTCATCAACACGTGTTTTCCT
TGGCCATGGA

CYTH1-EIF3H
ATGGAGGAGGACGACAGCTACG*ATGGCGTCCCGCAAGGAAGGTACCGGCTCTACTGCCACCTCTTCCAGC
TCCACCGCCGGCGCAGCAGGGAAAGGCAAAGGCAAAGGCGGCTCGGGAGAT

DHX35-ITCH
TACAGAGGGGCCAGGTGTAAGCATCTCTGAAGAGAGACAAAGTCTGGCTGAAAACTCTGGGACAACGGTTG
TTTACAACCCTTATGCTGCCCTTTCCATAGAGCAGCAGAGGCAGAAGCTGCCGGTATTCAAG*GTACCATGC
ATTTCACAGTGGCCTTGTGGAGACAACGCCTTAACCCAA

NFS1-PREX1
CGCACTCTTCTATCAG*GTGGACTCCATCAATGCTCTCCTCAAGGGGCCAGTCATGAGCCGGGCTTTCGAAG
AGACCAAGCATTTCCCTATGAACCACAGC
