# Supplementary material for: Identification of fusion genes in breast cancer by paired-end RNA-sequencing
Source: Genome Biol. 2011 Jan 19;12(1):R6. doi: 10.1186/gb-2011-12-1-r6 (PMC3091304; doi:10.1186/gb-2011-12-1-r6)
Supplement: Additional file 9 — Primer sequences used in the study. [file gb-2011-12-1-r6-S9.PDF]

Additional file 6. Primer sequences used for fusion gene validation:

Fusion gene	Primer type	Sequence (5´ → 3´)
DIDO1-KIAA0406	Forward	GTTGGCGCCAGAGTCAAAAG
	Reverse	GAGCTGCTGGTCTTTGGTTC
RPS6KB1-SNF8	Forward	GAGGACATGGCAGGAGTGTT
	Reverse	CTAGCTGGTCCTCAGCCAAG
VAPB-IKZF3	Forward	CTAAGGAACATGGCGAAGGT
	Reverse	GAGCTTTTTCGTTTTGCCAC
ACACA-STAC2	Forward	GCTAGGAGGAATACCTGTGGG
	Reverse	CCGAGCGAAGGAAGAAGTT
ZMYND8-CEP250	Forward	GCCATCTTTTACTGCTGTTGG
	Reverse	ATGGCCTCCATTTCTGTCTG
RAB22A-MYO9B	Forward	GTATTGTGTGGCGGTTTGTG
	Reverse	CCTCTCACGATGCCACTCTC
SKA2-MYO19	Forward	CGAGATGTTGAGTGACAGC
	Reverse	CTCATTGGCGTAGTTGATGC
STARD3-DOK5	Forward	GTGGCTGACATGGAGCAG
	Reverse	TGTGATGTGCTGCCAGTAGG
LAMP1-MCF2L	Forward	GGTAACGCCGCTGTCTCTAA
	Reverse	CAGCTGCGACTTATCGATGT
GLB1-CMTM7	Forward	CGGCCTTTTATATGGGGAAC
	Reverse	CAGGTAAAAGGCGAGGATCA
CPNE1-PI3	Forward	TCGCCACTTCTTCTCTGGAT
	Reverse	GACCTTTGACTGGCTCTTGC
BSG-NFIX	Forward	TGCTGGTCTGCAAGTCAGAG
	Reverse	TGCAGGTTGAACCAGGTGTA
PPP1R12A-SEPT10	Forward	CAAAACCCCTGGCTTCTGTA
	Reverse	ACCAGCTGATCAGGCAAACT
NOTCH1-NUP21	Forward	GGTGAGACCTGCCTGAATG
	Reverse	TGCTTGGCTTGGTTAGCAG
RARA-PKIA	Forward	TCCTGAATCGAGCTGAGAGG
	Reverse	TTGGAGATGACCTTCAGATTTC
TATDN1-GSDMB	Forward	GCAGTCATGAGTCGCTTCAA
	Reverse	ATATTGCCGGTCGCTTTTC
CSE1L-ENSG00000236127	Forward	TAATGCCGCACTCTATGCAC
	Reverse	ATGAAGACGAGGCTGTGGAC
ANKHD1-PCDH1	Forward	CAGTGCAGGTCATGTGGAAG
	Reverse	GGTACATGTGCCTGTCATGG
CCDC85C-SETD3	Forward	TCCCTCATCCACCTACATCAG
	Reverse	AAGGGTAGTTTGTTGGCATGA
SUMF1-LRRFIP2	Forward	CTTCTGGGAAAGACCGAGTG
	Reverse	TCGTTCCAGTTCTCTCATGC
WDR67-ZNF704	Forward	GTGCATGCATCAGGGAAATA
	Reverse	CCATGGCCAAGGAAAACAC
CYTH1-EIF3H	Forward	ATGGAGGAGGACGACAGCTA
	Reverse	GCCATCTATCTGCACTTGCTT
DHX35-ITCH	Forward	TACAGAGGGGCCAGGTGTAA

	Reverse	TTGGGTTAAGGCGTTGTCTC
NFS1-PREX1	Forward	CGCACTCTTCTATCAGGTGGA
	Reverse	GCTGTGGTTCATAGGGAAATG
BCAS4-BSAC3	Forward	CCTCCTGATGCTGCTCGT
	Reverse	CTCCTCCGTGTGCTCCAT
ARFGEF2-SULF2	Forward	CAGGAGAGCCAGACCAAGAG
	Reverse	ACTTGCCAGTGAGGATGGAG
RPS6KB1-TMEM49	Forward	TGCCATGAAGGTGCTTAAAA
	Reverse	GCATAACTTTGTGCCATGGAG

Other primers used for the study:

GAPDH	Forward	CTTTGGTATCGTGGAAGGACTC
	Reverse	AGTAGAGGCAGGGATGATGTTC

Primers used for siRNA efficiency validation in addition to fusion specific primers:

NUP214 wt	Forward	TCCCTCAAGTGGTAAATGTGC
IKZF3 wt	Forward	TGCATGCCAAAGAAGAGATG

Primers used for genomic DNA amplifications:

MYO9B	Reverse	AGAGTCTTCCCCGAGTTCTACC
RARAg_f02	Forward	TGTCTGTGCACAAATGTGTTTGGATGTA
PKIAg_r02	Reverse	TGCCTTCTGCTACTCAGAGATCACTCACC
LAMP1g_f01	Forward	CGTCTGGTAACGCCGCTGTCTCTAAC
MCF2L_r01	Reverse	CAGCTGCGACTTATCGATGTAACCGTGT
GLB1_f01	Forward	ATCTTTCCACTGGACACTGAGGAT
CMTM7g_r01	Reverse	GTGACCACTTCAAAGTAGCTGTAGG
CPNE1g_f01	Forward	GACATTCGCCACTTCTTCTCTGGATTGA
PI3g_r01	Reverse	TGACTGGCTCTTGCGCTTTGACTTTATC

CCDC85C-SETD3 and WDR67-ZNF704 genomic DNA amplifications were done by nested PCR.
Nested primers:

CCDC85C_f01	Forward	CATTGCCCCTTAATGAGAGATTGTGTGT
CCDC85C-SETD3	Reverse	AAGGGTAGTTTGTTGGCATGA
WDR67-ZNF704	Forward	GTGCATGCATCAGGGAAATA
ZNF704_r01	Reverse	CCTCAGGAACAAAGGCACAGAGACA
