# Supplementary material for: Identification of fusion genes in breast cancer by paired-end RNA-sequencing
Source: Genome Biol. 2011 Jan 19;12(1):R6. doi: 10.1186/gb-2011-12-1-r6 (PMC3091304; doi:10.1186/gb-2011-12-1-r6)
Supplement: Additional file 10 — FISH probes used for validation. [file gb-2011-12-1-r6-S10.PDF]

Sample	Target gene	FISH clone ID
BT474	RPS6KB1	RP11-111G18
BT474	SNF8	RP11-463M16
BT474	VAPB8	RP11-109J9
BT474	IKZF3	RP11-62N23
BT474	STARD3	RP11-98J2
BT474	DOK5	RP11-112O17
KPL4	BSG	RP11-575H1
KPL4	NFIX	RP11-245A21
KPL4	NOTCH	RP11-1008C19
KPL4	NUP214	RP11-1069P6
SKBR3	TATDN1	RP11-110D15
SKBR3	GSDMB	RP11-610O22
SKBR3	CYTH1	RP11-72M9
SKBR3	EIF3H	RP11-1080M3
